# Automated identification of flagella from videomicroscopy via the medial axis transform

**DOI:** 10.1038/s41598-019-41459-9

**Published:** 2019-03-21

**Authors:** Benjamin J. Walker, Kenta Ishimoto, Richard J. Wheeler

**Affiliations:** 1 Wolfson Centre for Mathematical Biology, Mathematical Institute, University of Oxford, Oxford, OX2 6GG UK; 2 Graduate School of Mathematical Sciences, The University of Tokyo, Tokyo, 153-8914 Japan; 3 Nuffield Department of Medicine, University of Oxford, Oxford, OX3 7BN UK; 4 Sir William Dunn School of Pathology, University of Oxford, Oxford, OX1 3RE UK

## Abstract

Ubiquitous in eukaryotic organisms, the flagellum is a well-studied organelle that is well-known to be responsible for motility in a variety of organisms. Commonly necessitated in their study is the capability to image and subsequently track the movement of one or more flagella using videomicroscopy, requiring digital isolation and location of the flagellum within a sequence of frames. Such a process in general currently requires some researcher input, providing some manual estimate or reliance on an experiment-specific heuristic to correctly identify and track the motion of a flagellum. Here we present a fully-automated method of flagellum identification from videomicroscopy based on the fact that the flagella are of approximately constant width when viewed by microscopy. We demonstrate the effectiveness of the algorithm by application to captured videomicroscopy of *Leishmania mexicana*, a parasitic monoflagellate of the family *Trypanosomatidae*. ImageJ Macros for flagellar identification are provided, and high accuracy and remarkable throughput are achieved via this unsupervised method, obtaining results comparable in quality to previous studies of closely-related species but achieved without the need for precursory measurements or the development of a specialised heuristic, enabling in general the automated generation of digitised kinematic descriptions of flagellar beating from videomicroscopy.

## Introduction

**Figure 1:**
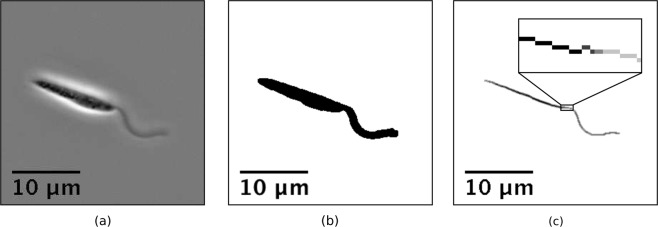
A sample frame taken from phase contrast videomicroscopy of a *L*. *mexicana* promastigote, from the dataset of Walker *et al*.^31^. (**a**) Original frame. Despite the presence of an accessory structure, the paraflagellar rod, at the recorded resolution the flagellum appears to be of approximately-constant width. (**b**) Result of processing (**a**) into a binary image, following background subtraction and noise reduction. Existing methods of flagellum extraction are unable to automatically identify the flagellum in this image, typically requiring user input at this stage. (**c**) Result of the medial axis transform applied to (**b**), encoding the width of the cell along the medial line. Shown inset is an enlarged section of the transform.

Well-studied and present across a wide range of organisms, the eukaryotic flagellum is typically a long slender organelle that is known to perform a variety of functional roles throughout nature, perhaps most notably in spermatozoa where the beating of one or more flagella can render a gamete motile^1^. Despite varying in function and also in lengthscale, conserved across eukaryotic flagella is the characteristic ‘9 + 2’ axoneme^2^. Formed of a central pair of singlet microtubules surrounded by nine microtubule doublets, this cytoskeletal structure is present along the length of the flagellum. This gives the organelle a well-defined diameter, although this may be obscured or complicated by the presence of accessory flagellar structures, such as the outer dense fibres of the mammalian spermatozoon^3^, or the paraflagellar rod present in the parasitic family *Trypanosomatidae*^4^. Despite structural additions to flagella, retained in typical optical videomicroscopy of flagellated organisms is an approximately constant flagellar width, as is exemplified in Fig. 1a.

Videomicroscopy of flagella at high resolution is pertinent to the study of many microswimmers, such as the classically-investigated *Crithidia oncopelti*^5^ and mammalian spermatozoon^6–10^, whether for use in examining motility or exploring the mechanics of a beating flagellum. In particular, obtaining a quantitative description of a flagellar beat constitutes a key part of, and a barrier to, further research, with significant human effort being dedicated to the problem. Researcher input ranges from early manual approaches akin to those of Ishijima *et al*.^7^ and Vernon and Woolley^11,12^, where flagellum tracing is done by hand using acetate overlays, to semi-automated or tailored methods^13–16^. The work of Wan *et al*.^13^ and Klindt *et al*.^15^ on the alga *Chlamydomonas* sees the flagellum extracted from experimentally-fixed cells using custom software, with Smith *et al*.^6^ utilising tuned image thresholding to ascertain the beating pattern of a spermatozoan flagellum, exploiting the high contrast between the spermatozoon body and flagellum specific to this microorganism and experimental setup. Present in each of these methods is a significant need for human input, be that in the calibration of a specialised algorithm or in the manual processing of each captured image. Thus there is scope for a fully-automated method for the identification and extraction of flagellar kinematics from videomicroscopy, and any such scheme would be of relevance to a wide scientific audience.

There are numerous semi and fully-automated methods for the digital tracking of filaments, capable of accurately describing the kinematics of even overlapping structures to sub-pixel precision, some examples being the commonly-used ‘FIESTA’ software^17^, the semi-automatic ‘Bohboh’ software (BohbohSoft, Tokyo, Japan)^18^, the generalised linear models of Xiao *et al*.^19^, and the ‘active contour’ methods of Goldstein *et al*.^20^, Hongsheng Li *et al*.^21^, Xu *et al*.^22^. Whilst invaluable in the tracking of free filaments, such methods are less applicable to the study of a moving flagellated microswimmer, where current methods may be unable to distinguish automatically between the swimmer body and any attached flagella, with the exception of specialised methods such as the probabilistic fitting approach of Yang *et al*.^23^ in application to spermatozoa. Figure 1a exemplifies a case where poor contrast between a flagellum and the rest of a cell may prevent typical thresholding methods from distinguishing between the cell and the flagellum, hence a different approach is needed to automatically differentiate between them.

Image transforms, such as the Hough and Radon transforms, are commonly used for feature extraction and image processing. A pertinent example, the Hough transform is used primarily as a method for detecting straight lines in binary images^24,25^, and has been applied to object tracking problems^26^, whilst the related Radon transform is widely used in tomographic reconstruction^27,28^. Less well-known than the Hough or Radon transforms, the medial axis transform has seen use in studies of cellular morphology^29,30^, with the transform encoding the approximate size and shape of image features. Owing to the noted characteristic shape of flagella, there is significant potential utility in this shape-encoding image transform in the context of flagellum tracking and identification.

Hence, in this paper we present a general fully-automatic method for the identification of flagella in preprocessed videomicroscopy, focussing in particular on the segmentation of an axoneme from a binary frame that includes a free-swimming non-axonemal body. We exploit a feature of the axoneme biology that is conserved across species and identifiable using the medial axis transform, and apply the resulting automatic segmentation algorithm to a large captured dataset of free-swimming *Leishmania mexicana* promastigotes, flagellated human pathogens of the family *Trypanosomatidae*. Evaluating our scheme against existing semi-automatic software, we then demonstrate the applicability of the proposed method to a spermatozoon dataset, showing multi-organism efficacy, and suggest and comment on a number of possible refinements of the automated scheme, considering also the performance of the scheme on reduced-quality datasets.

## Methods

### A common morphological trait

As touched upon in the introduction, the 9 + 2 cytoskeletal structure present across eukaryotic flagella defines the slender cylindrical morphology of the organelle^2^. The presence of this underlying structure along the entire length of the flagellum gives the filament a diameter that is approximately constant at typical optical microscopy resolutions, even when accessory structures are present. This is in stark contrast to what is in general the more-varied visible morphology of the cell body, as can be seen in Fig. 1, an example of a *L*. *mexicana* promastigote. Thus the flagellar morphology may distinguish the slender organelle from the remainder of a cell, and hence we will aim to isolate the flagellum from videomicroscopy by identifying this feature, recognising it as a region of consistent visible width.

### Medial axis transform

**Figure 2:**
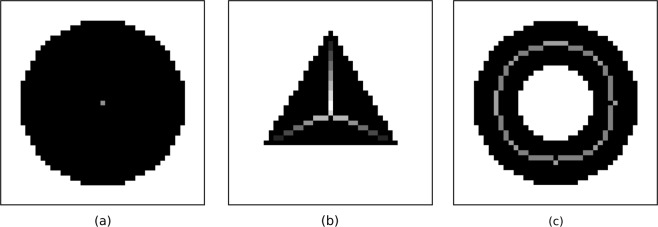
Examples of the medial axis transform applied to low-resolution simple shapes. The original image is shown in black, with the results of the transform superimposed in greyscale, where brighter pixels correspond to higher values of the transform and thus wider sections of the original shape. (**a**) A simple disk is mapped to a single point by the transform, with the radius of the disk encoded in the transform. (**b**) A triangular region is reduced to the skeleton shown, with the encoded width decreasing away from the skeleton and triangle’s shared centre. (**c**) An annulus is transformed to a circle along its medial line, with a constant width-profile subject to artefacts of the rasterisation.

In order to locate regions of constant width and subsequently identify them as axonemal filaments, we compute the medial axis transform of an image already processed into a binary representation. The medial axis transform may be thought of as the pixel-wise product of a Euclidean distance map and a skeletonisation, with the distance map encoding at each point the shortest distance to a point not included in the binary mask. As an example, consider the binary image shown in Fig. 1b, obtained via a prefiltering and image intensity thresholding of Fig. 1a, where the black region identifies the cell body and flagellum. Skeletonising the region gives a curve of the shape shown in Fig. 1c, where we are choosing to skeletonise such that the skeleton of a connected region is also connected. Taking the product of the resulting curve with a distance map of the original binary image yields the grayscale image of Fig. 1c, with points of greater magnitude (shown darker) corresponding to regions of greater width. Examples of the medial axis transform applied to simple shapes are shown in Fig. 2.

### Identifying the flagellar region

**Figure 3:**
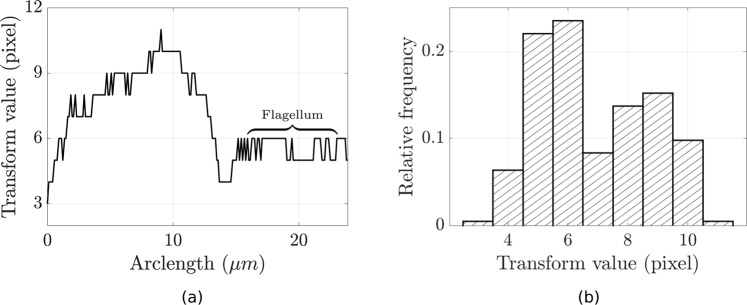
Analysis of a sample medial axis transform, corresponding to the cell in Fig. 1. (**a**) Values taken by the medial axis transform, shown in pixels, against the arclength, measured from left to right in Fig. 1. The flagellum may be clearly identified from this width-profile as the segment with approximately constant width, in contrast to the varied size of the rest of the cell. (**b**) A histogram of the discrete values of the transform shown in (**a**). A clear modal width can be seen around the flagellum width, suggesting that simple identification of the modal pixels may be sufficient to identify the flagellum in cases where the flagellar lengthscale is dominant.

The results of the medial axis transform may be used to identify a region of constant width, noted previously  to be a conserved morphological feature of flagella. Figure 3a shows an example width-profile of a flagellated swimmer, in particular the individual shown in Fig. 1. With arclength being measured along the computed skeleton starting from the left-most endpoint, a region of approximately constant width can be easily identified, corresponding precisely to the flagellum. Analysis of the derivatives of this profile with respect to arclength, for example examining the variation in the first derivative, can be used to isolate the point of flagellar attachment, and thus an endpoint of the flagellum, and subsequently to segment the entire flagellum by the identification of this constant-width region.

However, in many cases derivative analysis and sophisticated region identification from the width-profile may be an unnecessary complication. As indicated by the histogram of Fig. 3b, we note that the observed flagellar width found in Fig. 3a corresponds to the approximate mode of the width distribution of the cell. As the flagellum is typically a long slender organelle, it may be reasoned to be a significant contributing factor to a mode of the width distribution, and identified as such. Thus in cases where flagellar length is relatively large it is sufficient to simply identify those regions with cell width close to a modal value, subsequently taking care to select the largest of such regions. In particular, such modal analysis may be easily automated, and will be utilised in calculation.

### Procedure

Hence we propose that the tracing of flagella from a single frame may be achieved by the following overall procedure:

(i) Preprocess into a binary mask of the entire cell;
(ii) Perform and analyse a medial axis transform, isolating flagella by derivative or modal analysis;
(iii) Trace the resulting isolated filaments.

Both the preprocessing and filament tracing steps may be achieved automatically by use of existing tools, for example the methods of Ruhnow *et al*.^17^, Xiao *et al*.^19^ and Goldstein *et al*.^20^. The isolation of flagella via either the modal or derivative analysis suggested previously can also be perfomed without user input, with an example of a simplistic but effective automated modal analysis being provided in the Supplementary Information (Macros 1 and 2). Choosing a particular method of flagellar isolation prior to image processing, as we have done in Macros 1 and 2, yields a fully-automatic procedure for the identification of flagella from imaging data. Our presented automatic implementations make use of a simple effective modal analysis of the medial axis transform to accurately perform flagellar identification, additionally locating the point of flagellar attachment. This choice of implementation is motivated in the Appendices, where modal analysis can be seen to outperform a simple derivative-based approach.

Efficient and effective application of the above method requires datasets of suitable magnification, frame rate and contrast. In particular, magnification must be sufficiently high to sharply capture the flagella, typically with recorded flagellar width of at least 2/3 pixels. Frame rates should be high enough to avoid motion blur, and contrast levels such that the organism may be readily segmented from the background. Section 3.4 reports in detail on the performance of Macro 1 when applied to lower-quality imaging data, with an extension to datasets containing visually-overlapping flagella being briefly considered in the Appendices.

### Verification dataset

In order to verify the proposed algorithm we will process the dataset generated by Walker *et al*.^31^. Comprising of approximately 150,000 frames combined from 126 *Leishmania mexicana* promastigotes, in general the phase contrast videomicroscopy does not greatly differentiate the cell body from the attached flagellum, with the flagellum not having a different grey value to the cell body, thus existing thresholding approaches alone are not sufficient for the digital isolation of the flagellum.

### Evaluation metrics

To quantitatively assess the performance of any implementations against a baseline result we will use the measures of missed detection rate (MDR) and false detection rate (FDR) as used in the evaluation of the specialised sperm-tracing scheme of Yang *et al*.^23^. The MDR is the percentage of pixels present in the baseline result that are more than *d* pixels away from the segmented region produced by the test implementation, and represents the proportion of the true region that has not been identified by the test implementation. Conversely, the FDR is the percentage of pixels identified by the test implementation that are more than *d* pixels away from the baseline result, and represents the prevalence of falsely-identified regions. Here, following Yang *et al*.^23^, we will take *d* = 3 throughout unless otherwise stated, and note that an ideal implementation would result in the MDR and the FDR both being zero. We will also report on the proportion of frames in a sample in which subjects have been segmented successfully, where this is assessed qualitatively with reference to by-eye identification.

## Results

The proposed computational procedure was implemented in the ImageJ macro language^32^. Motivated by our findings in the Appendices, we opt in this instance to utilise the described modal analysis, creating a fully automated scheme that is computationally-trivial but proves to be effective in practice. Sample macros may be found in the Supplementary Information, where image preprocessing and flagellum tracing have been implemented in a basic manner that is sufficient for exemplifying the flagellar identification method.

### Flagellar identification in *L*. *mexicana*

The 150,000 frames of the dataset of Walker *et al*.^31^ were processed without user input using Macro 1 of the Supplementary Information, with the overall computational time (including preprocessing and flagellum tracing) being ca. 24 hours on a quad-core Intel Core i7-6920HQ CPU running ImageJ 1.51u, with peak memory usage no more than twice the size of a single frame.

**Figure 4:**
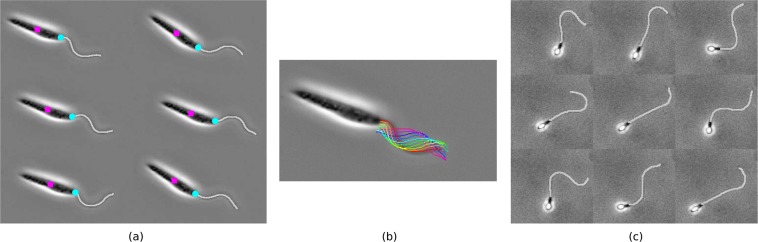
Composite data and results from an implementation of the proposed scheme. A montage of original frames with the identified flagella superimposed, showing very good agreement with identification by eye, for sample (**a**,**b**) *L*. *mexicana* and (**c**) spermatozoa. In (**a**) the computed cell centroids and locations of flagellar attachment are marked as magenta squares and cyan circles respectively. In (**b**) captured flagella from multiple frames are superimposed on the first frame of the sample dataset, with the simple sinusoidal nature of the flagellar beating being clearly visible. Original frames from the datasets of Walker *et al*.^31^ (unpublished) and Ishimoto *et al*.^37^ respectively. Reprinted original frames of (**b**) with permission from [K. Ishimoto, H. Gadêlha, E.A. Gaffney, D.J. Smith, J. Kirkman-Brown. Physical Review Letters 118, 124501, 2017] Copyright (2017) by the American Physical Society.

To demonstrate the efficacy of the algorithm a 100-frame sample of the results is presented in the Supplementary Information (Results 1), with the identified flagellum centreline being highlighted in white on the original sample (Dataset 1 of the Supplementary Information). In Fig. 4a we showcase multiple frames of this composite as a montage, with the flagellum centreline highlighted. Clear agreement can be seen between the segmented regions and what may be identified by eye as flagella, obtaining similar results to what may be expected of a manual tracing method but without any researcher input. The same level of accuracy is present in an overwhelming majority of analysed frames, with any loss only occuring due to the simplistic implementation of flagellum tracing used here. Thus we have validated both an implementation of and the methodology behind our proposed scheme of flagellar identification, in addition to enabling a quantitative study of *L*. *mexicana* flagellar kinematics.

Computed using the proposed automated method and as published by Walker *et al*.^31^, population-level beating statistics are given in Table 1 alongside those pertaining to the 100-frame *Leishmania* sample. Reported in detail by Walker *et al*., from the analysis of this dataset we may identify a characteristic sinusoidal form of the *Leishmania* flagellar beat. In particular, this sinusoidal form can be clearly seen in Fig. 4b, where captured flagella from multiple frames are shown superimposed and from which a simple travelling wave form may be identified. Indeed, as reported by Walker *et al*.^31^ Fourier analysis identifies this periodic beating as having only a single prominent temporal frequency, and thus a simple idealised functional form that enables data-driven kinematic studies of the swimming behaviours of *Leishmania*.

**Table 1:** Table of beat parameters extracted from the large *Leishmania* dataset of Walker *et al*.^31^.

Feature	Mean (±S.D.)	Sample
Dominant frequency	31.4 ± 9.42 Hz	29 Hz
Flag. wavelength	8.79 ± 1.99 μm	8.41 μm
Flag. amplitude	1.77 ± 0.877 μm	1.29 μm
Flag. length	14.9 ± 4.90 μm	10.6 μm

Beating characteristics are computed following application of the proposed automated analysis, with averages shown for the entire population. To illustrate the results of analysis for a single cell, the beat parameters of the 100-frame *Leishmania* sample provided in the Supplementary Information are also presented. Population-level statistics reproduced from Walker *et al*., 10.1016/j.jtbi.2018.11.016 under a Creative Commons Attribution License (http://creativecommons.org/licenses/by/4.0/CC BY), having been calculated via our proposed method of flagellar identification. With reference to the simple characteristic beating identified by Walker *et al*. using our proposed methodology, these features of the flagellar beating are sufficient to identify an idealised data-driven form of the *Leishmania* beat.

### Evaluation against an existing semi-automatic method

We evaluate the pixel-wise performance of our implemented mode-based method against the software package Bohboh (BohbohSoft, Tokyo, Japan)^18^, which uses an established semi-automatic method of flagellum segmentation^7,33–36^. The 100-frame *Leishmania* sample as used previously was processed using Bohboh, requiring approximately 15 minutes of continued researcher input and supervision, following which a TIFF containing the segmented flagellum data was reconstructed from the output. Processing of the same sample was also performed using Macro 1 of the Supplementary Information, which proceeded automatically without any researcher input and completed within 15 seconds using the computational resource already described.

Treating the reconstructed output of Bohboh as a baseline we compute the MDR and FDR as defined above, giving an MDR of 0.00% and an FDR of 1.92%. This minimal MDR corresponds to our implementation successfully identifying all of the regions found in the baseline, and hence correctly tracing the flagellum in each frame. The low FDR, comparable in magnitude to the 1.78% reported in the evaluation of the method of Yang *et al*.^23^, approximately represents the inclusion of 1–3 additional pixels per frame that are not present in the output of Bohboh, and thus further demonstrates significant agreement between the output of our implementation and that of Bohboh. Hence the results of our proposed fully-automated methodology and implementation are in strong agreement with the output of an established scheme. Further, our proposed method is approximately 60 times faster than using the semi-automatic software, and notably does not require any per-frame user input. Thus the proposed scheme is seen to give results comparable to existing methods, but with greatly-reduced user input and accompanying overall processing time.

### Application to a canonical flagellate

To highlight the applicability of the method and implementation to a variety of flagellated microorganisms we analyse a dataset of a swimming human spermatozoon, specifically Supplementary Movie 1 of Ishimoto *et al*.^37^. Example composite frames are shown in Fig. 4c, and as in the case of *L*. *mexicana* demonstrate remarkable accuracy, but we particularly note the presence of accessory structures to the flagellum. We see that the midpiece of the flagellum has consistently not been identified, owing to the greatly-increased width in this section, whilst the principal piece is accurately segmented. However, the midpiece is itself of approximately-constant visible width, thus we hypothesise that repeated modal analysis will be able to correctly identify and even distinguish between these different sections of the flagellum, and easily adapted from the current implementation.

Processing was performed automatically using Macro 2 of the Supplementary Material, which included slight adjustments to the preprocessing of Macro 1. In this example some sections of the flagellum briefly move out of the plane of imaging, which due to the simple preprocessing and filament tracing steps implemented here results in some errors in flagellum identification, in addition to variations in the final traced flagellum length. These are easily corrected by the use of more-sophisticated approaches to these stages of computation, such as those that have been previously commented upon above. As an additional example, reconstruction of a non-planar beating pattern may be performed as in Bukatin *et al*.^38^, where image intensity is used to infer the 3-dimensional location of swimming spermatozoa. Our approach may then be readily extended to 3 spatial dimensions.

### Performance on low-quality imaging data

**Figure 5:**
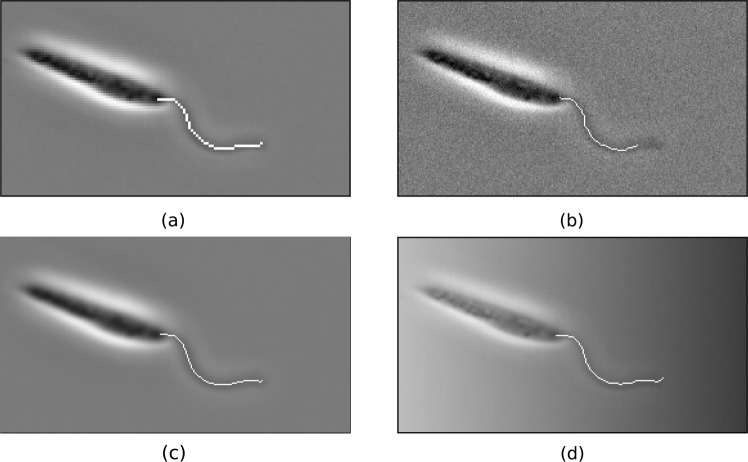
Composite degraded data and results from an implementation of the proposed scheme, where identified flagella are shown superimposed. Data has been degraded by application of: (**a**) downsampling (2x); (**b**) Gaussian noise; (**c**) Gaussian blur; (**d**) pixel-wise multiplication by a black-to-white horizontal gradient. Acceptable flagellar segmentation by Macro 1 of the Supplementary Material is seen for each degraded dataset shown here, with some loss of accuracy at the tip or base of the flagellum. In particular, Gaussian noise is seen in this case to prevent the distal tip of the flagellum being segmented from the image background, a result of the basic preprocessing implemented here and not characteristic in general of the proposed flagellum segmentation procedure.

In order to investigate the applicability of our methodology to a range of imaging data we evaluate the effectiveness of our implementation on intentionally-degraded datasets, emulating typical features of reduced-quality imaging. To duplicates of the 100-frame *Leishmania* sample introduced previously we perform a single image degradation, with examples shown in Fig. 5, and following the application of Macro 1 of the Supplementary Information to the dataset we compute the MDR and FDR relative to the baseline results established above.

The results of this testing are presented in Table 2 for a range of methods of image degradation mimicking common limitations of microscale imaging quality, where we have in turn applied downsampling, Gaussian noise, Gaussian blur, and gradient blending to the original sample image. When downsampling we reduce the resolution in each spatial dimension by a factor of 2 or 4, with successful segmentation still possible using Macro 1 in the case of a 2x reduction, whilst downsampling by a factor of 4 prevents reliable segmentation. Simulating higher framerate imaging, the effects of applied Gaussian noise of standard deviation 10 (relative to pixel values in the range 0–255) are minimal, with some loss of fidelity occurring near the distal flagellar tip. Applying a Gaussian blur of radius 2 to emulate subject defocussing or reduced resolution has little effect on the success of the segmentation, with Macro 1 performing well relative to by-eye comparison. Finally, blending the sample with a horizontal gradient to simulate non-uniform illumination results in segmentation of good accuracy, with a loss of precision around the flagellar attachment point that is expected to be improved by additional preprocessing. Given this consistent acceptable performance on all but the lowest quality data considered, the implementation presented in Macro 1 is evidenced to be robust to a variety of reasonable degradations in image quality, and hence is likely applicable to a range of imaging data without significant modification or additional preprocessing.

**Table 2:** Evaluated results of applying Macro 1 of the Supplementary Information to degraded imaging data.

Applied degradation	MDR (%)	FDR (%)	Success rate (%)
Downsampling (2x)	0.14	2.21	100
Downsampling (4x)	14.47	47.48	33
Gaussian noise	1.45	1.57	99
Gaussian blur	0.01	2.21	100
Gradient blend	0.02	6.12	100

Downsampling by a factor of 2 in each spatial dimension, Gaussian blur of radius 2, and blending with a horizontal gradient each do not prohibit successful flagellum segmentation via Macro 1, with some loss of attachment point fidelity in the case of the gradient blend. Gaussian noise causes a loss of accuracy in the distal flagellar region, but satisfactory segmentation is retained. Downsampling by a factor of 4 prohibits successful segmentation via this non-specialised implementation, with the resulting image having dimensions of only 68 × 38 pixels and hence being of very poor quality. When computing the MDR and FDR pertaining to the downsampled images *d* was taken to be 2 and 3 for the 2x and 4x downsampling respectively in order to account for the reduction in resolution.

## Discussion

In this work we have presented and verified a method of flagellar identification from the videomicroscopy of free-swimming bodies. The formulation of a general method optimised for all flagellated organisms or imaging contexts is not possible due to the diversity of flagellated life, hence we have presented a high-quality and robust method for addressing the basic case - that of a body with a single flagellum - which can be readily adapted to a broad range of organisms and contexts. We note the presence of a structural backbone ubiquitous in eukaryotic flagella and cilia, giving these axonemal filaments a well-defined cross-section and therefore a width. Additionally, we acknowledge that the presence of accessory structures to the axoneme may significantly alter the ultrastructure of the organelle, and therefore the width observed in videomicroscopy. However, the effects of some such structures have been considered, as for the cases of the paraflagellar rod of *Leishmania mexicana* and the outer dense fibres of particular spermatozoa, and change in visible width at typical optical resolutions and magnifications is either not significant in general or may be further exploited to identify subsections of a flagellum. Hence we proposed a procedure for the automated identification of flagella-like structures from videomicroscopy that exploited the morphological feature of consistent observed width, utilising the medial axis transform for quantification.

To extract the location of a flagellum from the results of the medial axis transform we have proposed two simple schemes: derivative analysis of the width-profile, where consideration of local extrema of the first and second derivatives is expected to be able to identify the proximal end of a flagellum, and a less-complex modal analysis of the width distribution. Having implemented and verified the latter approach, remarking that it is the most suitable of the two, we note the scope for future work to refine the precise methodology used here by performing more-sophisticated statistical analysis, however we emphasise that even the basic scheme shown in this work was sufficient for adequate identification of flagella.

Our approach is suitable for cases where an image can be binarised effectively, and we provide functional examples of preprocessing for typical samples of cells swimming in a background-free environment. However, any background subtraction or noise reduction approach could be used prior to using the algorithm described here. Successful identification of a flagellum here additionally depends upon the organelle being consistently contained within the focal plane, and may be less successful in processing datasets in which large out-of-plane deviations are consistently present - in the case of organism which uses an extensively 3-dimensional flagellum motion, a 2-dimensional video does not contain the necessary data for a complete analysis. Our approach is also likely unsuitable for use when processing numerous cilia/flagella that may not be easily distinguished, such as those of ciliated epithelia, but is in principle readily applicable to multi-flagellated microorganisms such as *Chlamydomonas*. In the Appendices we examined the potential effectiveness of the proposed methodology on datasets in which flagella are observed to cross one another, along with suggesting a suitable methodological refinement and thus demonstrating potential applicability to dense flagellate populations. This difficulty could be overcome in practice by diluting suspensions where possible.

Preprocessing and flagellum tracing were implemented here in a basic manner, and we recognise extensive scope for the incorporation of more-refined methods of filament segmentation, such as the ‘FIESTA’ suite of tools and active contour methods of Ruhnow *et al*.^17^ and Xiao *et al*.^19^ respectively, in addition to using established thresholding methods for the creation of an initial binary mask. However, despite the simplistic implementations used here, the proposed procedure was shown to provide desirable accuracy at little computational or human cost, enabling rapid quantification of flagellar motion in a sizeable dataset. Indeed, whilst the presented approach identifies flagella in individual frames, the achieved throughput is sufficiently high for use in video analysis, and has been demonstrated to be robust to reductions in image quality.

A potential refinement to the proposed scheme involves combining our morphological analysis with segmentation based on signal magnitudes, valuable in cases where thresholding-based segmentation may be informative but only partially applicable. We also suggest an application to the tracking of free-swimming microorganisms, where the analysis of the width-profile may yield the location of flagellar attachment and thus enable the tracking of swimmer motion.

In summary, our proposed method for the identification of axonemal filaments has been demonstrated to be of high accuracy and able to be implemented as a fully-automated algorithm. We have verified our method on a large dataset captured of a free-swimming flagellate, and achieved a high throughput and accuracy without optimisation or refinement of a basic implementation. Notably reliant only on a conserved morphological feature of axonemal filaments, the considered procedure and implementations may potentially realise future study of the kinematics of a wide range of flagellated and ciliated microorganisms.

## Comparison of Modal and Derivative Analysis

We consider the viability of the derivative analysis suggested in the main text, evaluating a simple implementation on the sample *Leishmania* dataset provided in the Supplementary Information and comparing the results against a modal analysis of the same width-profile. We will evaluate performance on the simplest example of a flagellated swimmer, that with a single flagellum that does not appear to intersect itself, with each method’s performance on this test case expected to be indicative of its performance in more general settings. The modal analysis is implemented as in Macros 1 and 2 of the Supplementary Information, where the mode of the width-profile is identified and then used as a simple threshold to isolate the image regions corresponding to the flagellum.

Presented in Macro 3 of the Supplementary Information is a scheme based on a simple derivative analysis, where the resulting skeleton of the medial axis transform is traced from the flagellar tip and an approximation to the derivative of the width-profile is computed via a forward difference operator. At each point in the transform we determine if the approximated derivative is an outlier in the context of the previously-computed derivatives, allowing for noise due to the rasterised image and the discrete nature of the width-profile and skeleton. Informed by the profile of Fig. 3a, such an outlier is assumed to correspond to the point of flagellar attachment, at which we will have completely traced the portion of the skeleton corresponding to the flagellum. This method of analysis is configurable via tuning of the foward difference operator and the details of outlier identification, with a 3-step forward difference operator being used here, found to give optimal segmentation results for this test dataset.

On the 100-frame sample *Leishmania* dataset both methods of flagellum identification achieve comparable pixel-wise accuracy to by-eye segmentation, with the derivative-based method more-faithfully tracing the skeleton of the medial axis transform than the modal thresholding method but the resulting difference in output being less than 1% of pixels, with typically only a single pixel being affected. Though the derivative analysis gives a marginal increase in pixel-wise accuracy over our implemented modal analysis, on the sample dataset the derivative method correctly segments only 97% of the given frames, in comparison to the 100% success rate of the modal analysis. Further, the success of the derivative-based scheme is seen to be highly sensitive to the details of the derivative approximation and method of outlier detection, with the use of 2-step and 4-step foward difference operators correctly identifying the approximate point of flagellar attachment in only 71% and 95% of frames respectively. This sensitivity is expected to result in reduced general applicability of the derivative-based approach in comparison to the modal analysis, the latter having no comparable configurable parameters and noted to perform satisfactory automated segmentation.

Thus we conclude that a simple modal analysis is better suited for application to the test dataset than our derivative-based approach, with the robustness and frame coverage of the former outweighing slight increases in pixel-wise accuracy afforded by our derivative-based method. We therefore expect a modal approach to be of the most utility in analysing large and diverse datasets, where a robust and reliable method may be preferable to a highly-sensitive scheme with marginally-improved accuracy.

## Adaptation to Visually-Overlapping Flagella

Unseen in our analysed datasets but more prevalent in general, overlapping flagella present possible complications to the application of our proposed method of flagellum segmentation and tracing, requiring additional consideration or more-sophisticated tracing methods. Here we briefly discuss adaptations of the presented method to address two such complications, though we emphasise that full consideration of complex flagellar interactions requires significant future study.

### Effects on the observable width

The apparent crossing of two flagella may intuitively be thought to increase the observable width of any binary representation at the crossing point, correspondingly increasing the value encoded in the medial axis transform around that point. From manual examination of a range of artificial flagellar intersections we remark that the crossings do not appear to significantly increase the value of the transform in a large number of cases.

However, large variation may be observed when intersections are approximately tangential, potentially doubling the observable width encoded in the transform. Such cases may render the flagella indistinguishable from one another, but in more-favourable cases will simply cause a large local increase in the values of the transform. The modal implementation proposed in this study would then typically result in disconnected components of the transform, and thus would fail to identify the entire flagellar region.

We propose a simple augmentation to the existing methodology: the linear stitching of any nearby isolated segments of the thresholded transform to restore connectivity. Limiting the radius of reconnection to the approximate flagellum width prevents the closure of any properly-segmented regions, whilst enabling the joining of regions of flagellum overlap. Initial evaluation of a simple implementation of this refinement step on artificial test cases gives significantly improved accuracy, enabling segmentation of a range of crossing scenarios. However, this scheme is unable to reliably segment approximately-tangential crossings, with future work needed to address this.

### Ill-defined transform arclength

Crossing and self-intersection of flagella results in an ill-defined transform arclength due to branching, potentially of issue to flagellum segmentation and tracing. However, the considered and preferred modal analysis is independent of transform topology and arclength, and thus only the post-processing step of flagellum tracing is affected. In Macro 4 of the Supplementary Information we provide a simple adaptation of the simple tracing procedure used previously, resolving branches in the post-segmentation skeleton by attempting to preserve the local flagellum tangent, appropriate for all but the highest-curvature scenarios. We provide sample test cases in Datasets 2 and 3 of two crossing skeletons, which are correctly traced by Macro 4. Refinements of this sample approach may include curvature-preserving branch resolution, though such work represents a large field in image analysis research.

## Supplementary information

Supplementary Information

Supplementary Datasets and Macros

## Data Availability

The full datasets generated during and/or analysed during the current study are available from the corresponding author on reasonable request. Sample codes and datasets are included in this published article (and its Supplementary Information files).
