# Supplementary material for: Automated identification of flagella from videomicroscopy via the medial axis transform
Source: Sci Rep. 2019 Mar 21;9:5015. doi: 10.1038/s41598-019-41459-9 (PMC6428899; doi:10.1038/s41598-019-41459-9)
Supplement: Supplementary file 1 — Supplementary Information [file 41598_2019_41459_MOESM1_ESM.pdf]

Automated identification of flagella from videomicroscopy via the medial axis transform
-
Supplementary Information

Benjamin J. Walker, Kenta Ishimoto and Richard J. Wheeler.

- Macro 1 - `find_flag_leish.txt`. A macro written in the ImageJ macro language for the automated extraction of flagella from Dataset 1.
- Macro 2 - `find_flag_sperm.txt`. A macro written in the ImageJ macro language for the automated extraction of flagella from the dataset of Ishimoto et al. (2017), an example of a free-swimming human spermatozoa. Differs from Macro 1 only in preprocessing.
- Macro 3 - `find_flag_leish_deriv.txt`. A macro written in the ImageJ macro language for the automated extraction of flagella from Dataset 1 using derivative analysis for flagellum identification.
- Macro 4 - `trace_smooth_path.txt`. A test macro demonstrating simple filament tracing for visually-intersecting filaments. For use on Dataset 2 and Dataset 3.
- Dataset 1 - `leish_sample.tif`. A 100-frame sample of captured phase contrast videomicroscopy of a free-swimming *Leishmania mexicana* promastigote, from the unpublished dataset of Walker et al. (2019), published in part here with the author's permission.
- Dataset 2 - `crossing_skeletons1.tif`. A sample frame of a skeleton with intersections, for use with Macro 4.
- Dataset 3 - `crossing_skeletons2.tif`. A sample frame of a skeleton with intersections, for use with Macro 4.
- Results 1 - `leish_composite.tif`. The results of Macro 1 applied to Dataset 1, shown superimposed on the original dataset.

References

Ishimoto, K., Gadêlha, H., Gaffney, E.A., Smith, D.J., Kirkman-Brown, J., 2017. Coarse-graining the fluid flow around a human sperm. Physical Review Letters 118, 124501. doi:`10.1103/PhysRevLett.118.124501`.

Walker, B.J., Wheeler, R.J., Ishimoto, K., Gaffney, E.A., 2019. Boundary behaviours of Leishmania mexicana: A hydrodynamic simulation study. Journal of Theoretical Biology 462, 311 – 320. doi:`10.1016/j.jtbi.2018.11.016`.
