# Supplementary figures and images for: Automated identification of flagella from videomicroscopy via the medial axis transform
Source: Sci Rep. 2019 Mar 21;9:5015. doi: 10.1038/s41598-019-41459-9 (PMC6428899; doi:10.1038/s41598-019-41459-9)

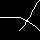

Supplement: Supplementary file 2 — Supplementary Datasets and Macros [file 41598_2019_41459_MOESM2_ESM.zip › crossing_skeletons1.tif]

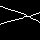

Supplement: Supplementary file 2 — Supplementary Datasets and Macros [file 41598_2019_41459_MOESM2_ESM.zip › crossing_skeletons2.tif]

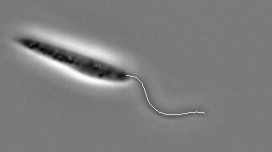

Supplement: Supplementary file 2 — Supplementary Datasets and Macros [file 41598_2019_41459_MOESM2_ESM.zip › leish_composite.tif]

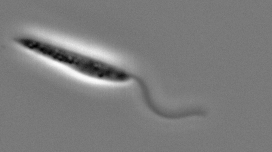

Supplement: Supplementary file 2 — Supplementary Datasets and Macros [file 41598_2019_41459_MOESM2_ESM.zip › leish_sample.tif]
